# A three-component monooxygenase from *Rhodococcus wratislaviensis* may expand industrial applications of bacterial enzymes

**DOI:** 10.1038/s42003-020-01555-3

**Published:** 2021-01-04

**Authors:** Makoto Hibi, Dai Fukuda, Chihiro Kenchu, Masutoshi Nojiri, Ryotaro Hara, Michiki Takeuchi, Shunsuke Aburaya, Wataru Aoki, Kimihiko Mizutani, Yoshihiko Yasohara, Mitsuyoshi Ueda, Bunzo Mikami, Satomi Takahashi, Jun Ogawa

**Affiliations:** 1 Biotechnology Research Center and Department of Biotechnology, Toyama Prefectural University, 5180 Kurokawa, Imizu, Toyama 939-0398 Japan; 2 Laboratory of Industrial Microbiology, Graduate School of Agriculture, Kyoto University, Kitashirakawa-oiwakecho, Sakyo-ku, Kyoto, 606-8502 Japan; 3 Division of Applied Life Sciences, Graduate School of Agriculture, Kyoto University, Kitashirakawa-oiwakecho, Sakyo-ku, Kyoto, 606-8502 Japan; 4 Biotechnology Research Laboratories, Kaneka Corporation, 1-8 Miyamae, Takasago, Hyogo, 676-8688 Japan

**Keywords:** Biocatalysis, Oxidoreductases

## Abstract

The high-valent iron-oxo species formed in the non-heme diiron enzymes have high oxidative reactivity and catalyze difficult chemical reactions. Although the hydroxylation of inert methyl groups is an industrially promising reaction, utilizing non-heme diiron enzymes as such a biocatalyst has been difficult. Here we show a three-component monooxygenase system for the selective terminal hydroxylation of α-aminoisobutyric acid (Aib) into α-methyl-D-serine. It consists of the hydroxylase component, AibH1H2, and the electron transfer component. Aib hydroxylation is the initial step of Aib catabolism in *Rhodococcus wratislaviensis* C31-06, which has been fully elucidated through a proteome analysis. The crystal structure analysis revealed that AibH1H2 forms a heterotetramer of two amidohydrolase superfamily proteins, of which AibHm2 is a non-heme diiron protein and functions as a catalytic subunit. The Aib monooxygenase was demonstrated to be a promising biocatalyst that is suitable for bioprocesses in which the inert C–H bond in methyl groups need to be activated.

## Abstract (web summary)

Makoto Hibi et al. report a novel three-component monooxygenase system in *Rhodococcus wratislaviensis*. This enzyme catalyzes the activation of an inert C–H bond and may be potentially important as a biocatalyst for industrial applications.

## Introduction

α-Aminoisobutyric acid (Aib) is a non-proteinogenic amino acid and acts as a strong helix inducer in peptides. Aib has attracted interest from an enzymological perspective because it lacks an α-hydrogen atom. Abstraction of the α-hydrogen atom from α-amino acids is a crucial step for many amino acid converting enzymes^1–3^. α,α-Dialkyl α-amino acids, such as Aib, are considered to have existed on Earth during the ancient ages because they are present as extra-terrestrial amino acids in carbonaceous chondrite meteorites^4,5^ and micrometeorites^6^. It is estimated that micrometeorites deliver about 2–8 × 10^5^ g of Aib to the Earth’s surface per year^6^. Aib in the biosphere can serve as a component of peptide antibiotics produced by several genera of fungi^7,8^. The only enzyme known to react with Aib is α,α-dialkylglycine decarboxylase^9^, which is a pyridoxal-5′-phosphate (PLP)-dependent enzyme that converts Aib into acetone with the release of carbon dioxide and also transfers the α-amino group from Aib to pyruvic acid to produce l-alanine (l-Ala).

Here, we discovered a three-component monooxygenase system consisting of AibH1H2, AibG, and AibF for Aib hydroxylation in a *Rhodococcus wratislaviensis* strain possessing an oxygen-dependent Aib catabolic pathway not dependent on α,α-dialkylglycine decarboxylase.

Dioxygen plays an important role in natural processes of metabolism and detoxification, and the activation of dioxygen by metalloenzymes has attracted chemical and biochemical interest. Non-heme diiron enzymes, which form a high-valent iron-oxo species at the diiron center, are a remarkable group of such metalloenzymes^10^. The non-heme diiron enzymes activate dioxygen at ambient temperature and pressure to catalyze many reactions involved in important biological processes. These processes include: methane hydroxylation in methanotrophs; the degradation of aromatic compounds; the biosynthesis of deoxyribonucleotides, fatty acids, and antibiotics; and the regulation of cell proliferation^11^. Their ability to selectively hydroxylate a variety of hydrocarbon substrates, such as alkanes and aromatics has important practical applications. The direct and selective functionalization of inert C–H bonds is useful for fuel production and industrial biosynthetic applications by improving step-economy and atom-economy^12,13^.

AibH1H2, a non-heme diiron enzyme, acts as the terminal oxygenase in the three-component monooxygenase system to produce α-methyl-d-serine (D-MeSer). All previously reported amino acid hydroxylases that form hydroxy groups on an aliphatic C–H bond belong to the Fe(II)/α-ketoglutarate-dependent dioxygenase (2OGDO) superfamily^14–16^, but AibH1H2 belongs to the amidohydrolase superfamily (AHS). We characterized the Aib monooxygenase through biochemical and structural analyses, and illustrate that it is an active biocatalyst for the inert C–H bond in methyl groups, thereby expanding the potential applications of such enzymes in industrial bioprocesses.

## Results

### Screening and catabolite analysis of Aib-degrading microorganisms

Approximately 2500 soil microorganism strains were isolated from an enrichment culture with Aib as the sole carbon source. Among them, seven strains exhibited the ability to degrade Aib in the resting cell reactions. Similar to previous reports^17,18^, four of these isolates produced acetone through Aib degradation. These acetone-forming Aib-degrading strains possess PLP-dependent α,α-dialkylglycine decarboxylase activity because the Aib degradation and acetone production were completely inhibited by aminooxyacetic acid (AOA), a well-known PLP-dependent enzyme inhibitor^19^. In contrast, the other three isolates, C31-06, C63-06, and C86-07, did not produce acetone, and Aib degraded even in the presence of AOA. Our results indicate that these microorganisms possess an undiscovered Aib catabolic pathway that is independent of the α,α-dialkylglycine decarboxylase reaction. Based on 16S ribosomal DNA sequence analysis, isolates C31-06 and C63-06 were found to be most closely related to *Rhodococcus wratislaviensis*, and C86-07 to *Nocardia globerula*. *R. wratislaviensis* C31-06, which showed the most active growth on Aib, was selected for further analysis.

**Fig. 1:**
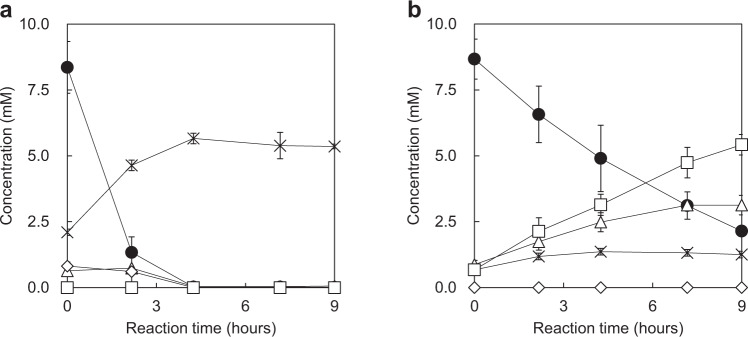
Analysis of Aib catabolites from *R. wratislaviensis* C31-06. Time-course of catabolite concentrations, including Aib (filled circles), D-MeSer (empty triangles), Amma (empty squares), l-Ala (empty diamonds), and ammonia (crosses) detected in resting cell reactions **a** without AOA and **b** with 1.0 mM AOA. Data represent the mean of three independent experiments.

The Aib catabolites were identified based on their molecular masses and retention times in liquid chromatography-mass spectrometry (LC-MS) analysis corresponding to the values for standard substances. During the resting cell reaction of *R. wratislaviensis* C31-06, several catabolites, including ammonia and alanine, were detected early in the reaction but ammonia was the only Aib catabolite detected after 4 h of the reaction (Fig. 1a). However, in the presence of AOA, two additional compounds accumulated in the resting cell reaction (Fig. 1b). One of these compounds were identified as α-methylserine.  A chiral high-performance liquid chromatography (HPLC) analysis of α-methylserine and alanine formed in the reaction showed that D-MeSer and l-Ala were preferentially formed. The second Aib catabolite formed in large amounts had a molecular mass 30 Da higher than that of Aib. This catabolic product was purified from the reaction mixture and nuclear magnetic resonance (NMR) spectroscopy analysis identified it as 2-amino-2-methylmalonic acid (Amma) based on a comparison with data previously reported in relevant literature^20^. From the above results, a novel pathway for Aib catabolism by *R. wratislaviensis* C31-06 was proposed. In this pathway, Aib is initially converted into D-MeSer and then oxidized to Amma. Subsequently, Amma is degraded to l-Ala, which is followed by ammonia formation. Amma was accumulated only when AOA was added to the reaction, which indicates that a PLP-dependent enzyme is involved in the degradation of Amma into ammonia. Additionally, the conversion of Aib to D-MeSer was only detected in the cells grown in culture medium containing Aib as the sole carbon source (Supplementary Fig. 1). Thus, Aib catabolism in *R. wratislaviensis* C31-06 is regulated by Aib.

### Identification of the Aib-hydroxylating enzyme in *R. wratislaviensis* C31-06

Proteomic profiles of *R. wratislaviensis* C31-06 cultivated under Aib-induced and non-induced conditions were investigated through a quantitative comparative proteome analysis using liquid chromatography-tandem mass spectrometry (LC-MS/MS)^21^ and followingly compared. The proteins induced in the presence of Aib with log_2_ fold-change > 1 and *p*-value < 0.01 were identified (Supplementary Fig. 2). Some of the highly-induced proteins were encoded by genes that form a gene cluster (Rhow_000797–000808) in the *R. wratislaviensis* C31-06 genome (Table 1). Most of these proteins are annotated as enzymes related to amino acid metabolism. Therefore, this gene cluster is considered to be responsible for Aib catabolism and is termed the “*aib* gene cluster”. Except for AibR—a LysR family transcriptional regulator that was not detected in the proteome analysis—all proteins encoded in this cluster were strongly induced by Aib.

**Table 1:** The *aib* gene cluster in *R. wratislaviensis* C31-06.


Gene ID	Gene	Protein size (amino acids)	logFC	BLASTP search		Function elucidated in this study
				Ortholog	Identity/Similarity (%)	
*Rhow_000808*	*aibR*	279	n.a.	LysR family transcriptional regulator [*Saccharopolyspora spinosa*]	62/77	Putative transcriptional regulator
*Rhow_000807*	*aibD1*	300	2.35	3-Hydroxyisobutyrate dehydrogenase [*Rhodococcus* sp. Leaf7]	66/77	NAD^*+*^-dependent D-MeSer dehydrogenase
*Rhow_000806*	*aibE*	491	2.31	Aldehyde dehydrogenase [*Saccharopolyspora spinosa*]	81/90	NAD^*+*^-dependent Amms dehydrogenase
*Rhow_000804*	*aibH1*	373	2.24	Amidohydrolase [*Rhodococcus* sp. 06-418-1B]	77/87	Aib hydroxylase, α' subunit
*Rhow_000803*	*aibH2*	378	2.57	Amidohydrolase [*Rhodococcus*]	75/86	Aib hydroxylase, α subunit
*Rhow_000802*	*aibG*	169	2.09	Rieske (2Fe-2S) protein [*Pseudonocardia* sp. P1]	62/79	Ferredoxin
*Rhow_000801*	*aibF*	322	2.18	2Fe-2S iron-sulfur cluster binding domain-containing protein [*Streptomyces* sp. SID8352]	67/81	Ferredoxin:NAD^*+*^ oxidoreductase
*Rhow_000800*	*aibT*	480	3.20	Amino acid permease [*Rhodococcus opacus*]	84/89	Aib/D-MeSer transporter
*Rhow_000799*	*aibC*	452	2.20	Serine hydroxymethyltransferase [*Rhodococcus opacus*]	74/83	Amma decarboxylase
*Rhow_000798*	*aibA*	366	2.32	Alanine dehydrogenase [*Rhodococcus opacus*]	91/95	NAD^*+*^-dependent l-Ala dehydrogenase
*Rhow_000797*	*aibN*	465	1.28	NAD(P)/FAD-dependent oxidoreductase [*Rhodococcus jostii*]	82/88	Function unknown

The cluster (Rhow_000797–000808) includes eleven protein-encoding genes relevant to Aib catabolism. The *aibH1H2GF* gene set, encoding a three-component Aib monooxygenase system, is shown by black arrows.

*n.a*. not applicable, *logFC* log_2_ (fold change of Aib-induction).

**Fig. 2:**
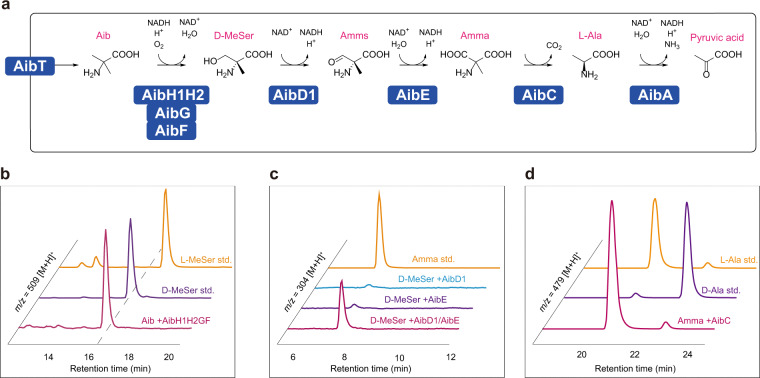
Elucidated Aib-catabolic pathway in *R. wratislaviensis* C31-06. **a** Aib is degraded into pyruvic acid via a five-step enzymatic reaction. **b** A chiral HPLC analysis of D-MeSer produced from reactions catalyzed by AibH1H2GF. The *m/z* 509 [M+H]^+^ represents a mass to charge ratio of d/l-MeSer derivatized with GITC. **c** Analyses of products from the reactions catalyzed by AibD1 and AibE. AccQ derivative of Amma indicated *m/z* 304 [M+H]^+^. **d** Stereoselective Amma decarboxylation activity of AibC. Alanine formed in the Amma decarboxylation reaction mixtures was analyzed by chiral amino acid analysis. The *m/z* 479 [M+H]^+^ represents a mass to charge ratio of d/l-Ala derivatized with GITC.

Hydroxylation is the most likely reaction for the conversion of Aib to D-MeSer. However, the proteins encoded in the *aib* gene cluster bear no similarity to 2OGDO or any other common oxygenase such as cytochrome P450 monooxygenase (CYP)^22^ or flavin-dependent monooxygenase^23^. Two genes in the *aib* cluster, *aibH1* and *aibH2*, are located just upstream of the *aibF* and *aibG* genes annotated as a Rieske-type ferredoxin and a [2Fe-2S] binding domain-containing protein, respectively. Therefore, AibH1 and AibH2 are likely to act as terminal oxygenase components in the Aib hydroxylation system by accepting electrons from redox partner proteins AibG and AibF similar to the electron transfer chain of Class I CYPs^24^. Both AibH1 and AibH2 are amidohydrolase superfamily (AHS) proteins with 28% amino acid identity to each other. Hence, a recombinant *Rhodococcus erythropolis* strain was constructed to express AibH1, AibH2, AibG, and AibF (AibH1H2GF), and was followingly assessed for its Aib hydroxylation activity. As expected, D-MeSer was produced from Aib by the recombinant strain (Fig. 2b) with a specific activity of 8.33 nmol^−1^ min^−1^ ml of culture^−1^. The hydroxylation reaction by AibH1H2GF was strictly stereoselective, and only D-MeSer was obtained in the culture supernatant without the by-product of L-MeSer. No D-MeSer production was observed in cultures of the host strain or other recombinant strains expressing incomplete combinations of these four proteins, i.e., AibH1GF, AibH2GF, AibH1H2F, or AibH1H2G.

AibF was heterologously expressed for further enzymatic characterization. The absorption spectrum of the cofactor released from purified AibF showed maxima at 370 and 446 nm (Supplementary Fig. 3a), which indicates that the enzyme contains a flavin cofactor^25^. In HPLC analysis of the cofactor released from AibF, flavin mononucleotide (FMN) was detected as a major peak (Supplementary Fig. 3b). Therefore, noncovalently bound FMN was identified as a cofactor of AibF. AibF showed diaphorase activity with much higher affinity for NADH than for NADPH, with a specific activity of 271 μmol^−1^ min^−1^ mg^−1^. Thus, AibF was found to be a ferredoxin:NAD^+^ oxidoreductase containing FMN.

**Fig. 3:**
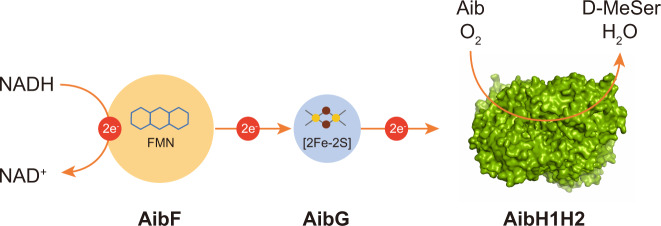
The three-component enzyme system used for the hydroxylation of Aib to produce D-MeSer in *R. wratislaviensis* C31-06. The electron pair of NADH is transferred to the hydroxylase component (AibH1H2) via electron transfer proteins (AibF and AibG). AibH1H2 catalyzes the hydroxylation of Aib to produce D-MeSer together with water, by using the received electron pair and molecular oxygen. AibF, ferredoxin:NAD^+^ oxidoreductase; AibG, ferredoxin; AibH1H2, α_2_α′_2_ heterotetrameric Aib hydroxylase.

AibH1 and AibH2 likely form a tetrameric complex (Supplementary Fig. 4a) and were tightly bound in approximately equal amounts (Supplementary Fig. 4b). Thus, AibH1 and H2 are α′ and α subunits forming an α_2_α′_2_ heterotetrameric complex that act as the hydroxylase component in the Aib monooxygenase. Overall, AibH1H2GF forms a three-component (hydroxylase (AibH1H2), ferredoxin (AibG), and ferredoxin:NAD^+^ oxidoreductase (AibF)) system transferring electrons from NADH to the terminal oxygenase to catalyze stereoselective hydroxylation of Aib to form D-MeSer (Fig. 3).

### Structure of the AibH1H2 hydroxylase complex

**Fig. 4:**
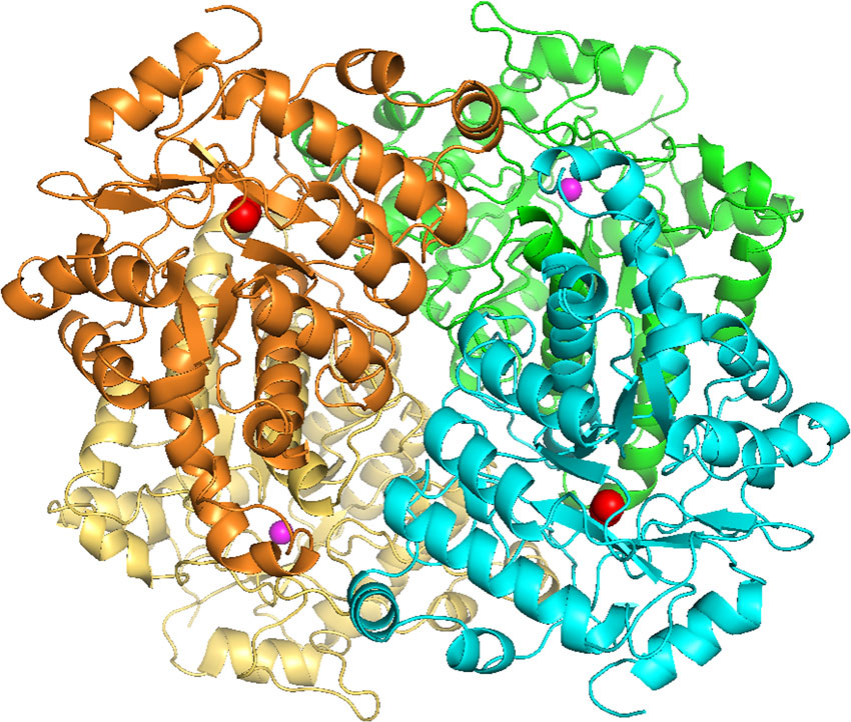
Overall structure of AibH1H2 complex. Ribbon diagram of an α_2_α′_2_ heterotetrameric unit of two AibH1 subunits (yellow and green) and two AibH2 subunits (orange and cyan) are shown. Pink and red spheres are Zn and Fe ions, respectively.

**Fig. 5:**
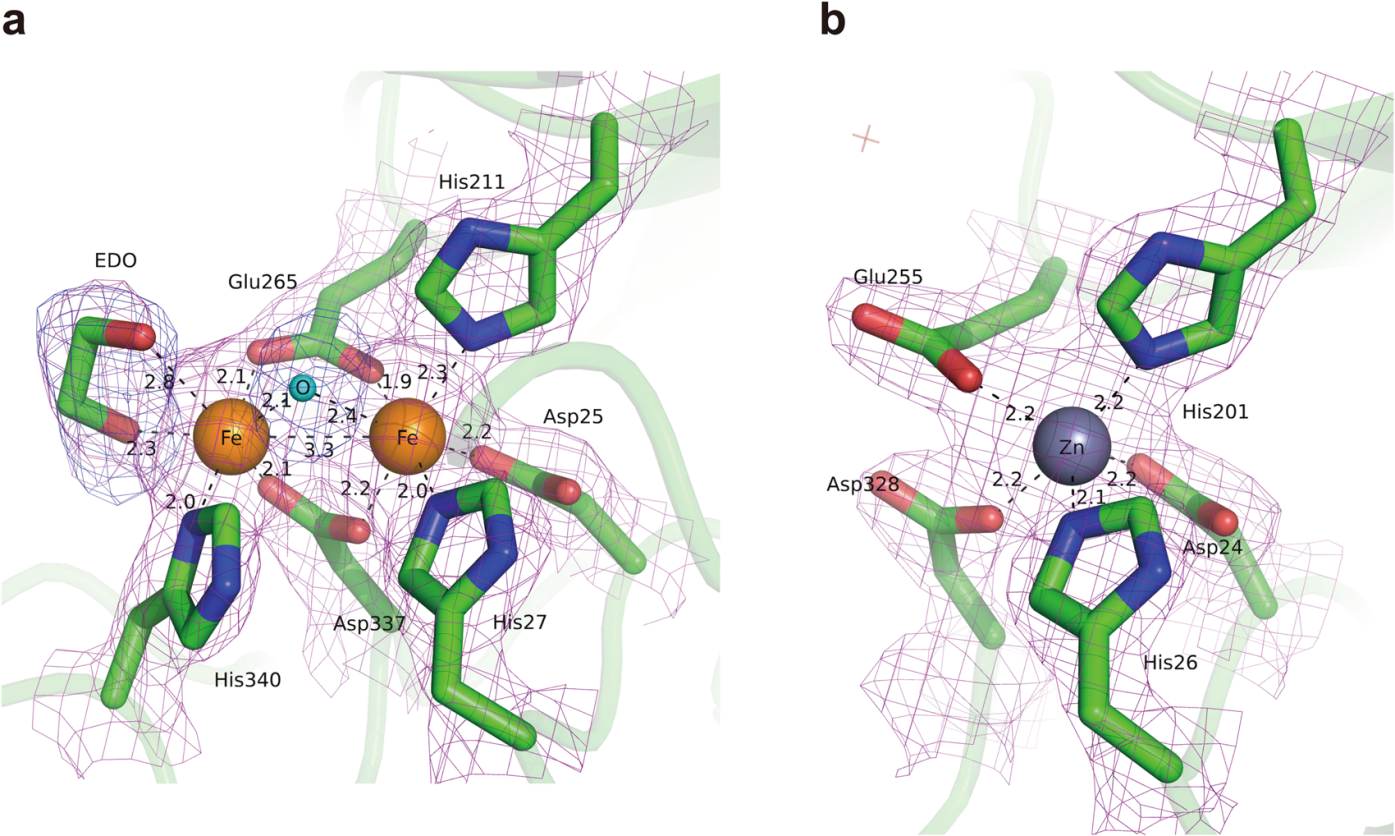
Overview of the metal-binding sites in AibH1H2 complex. The (2*F*_o_ − *F*_c_) OMIT electron-density map (magenta mesh) contoured at 1.4 σ is superimposed on the refined model. **a** AibH2 subunit bound to two Fe ions. Fe ions are shown as orange spheres and coordinated by six AibH2 residues (shown as the green sticks), O atom (cyan sphere), and EDO. The (*F*_o_ − *F*_c_) OMIT electron-density map (blue mesh) contoured at 3.0 σ is also superimposed. **b** AibH1 bound to a Zn ion. Zn ion is shown as a gray sphere and coordinated by five AibH1 residues (shown as the green sticks). Black dashes indicate metal coordination and selected hydrogen bonds.

The crystal structure of AibH1H2 hydroxylase was determined to a resolution of 2.45 Å (Table 2). AibH1H2 has a heterotetrameric structure of AibH1 and AibH2 subunits, and each subunit has the characteristic (β/α)_8_-barrel (TIM-barrel) fold present in all members of the AHS (Fig. 4). The AibH1 and AibH2 subunits interact via 36 hydrogen bonds and numerous van der Waals interactions (Supplementary Fig. 5). The structures of AibH1 and AibH2 align well displaying a root-mean-square deviation of 1.75 Å for 294 residues, despite the overall amino acid identity of just 28% (Supplementary Fig. 6). Clear electron density is observed in the crystal structures of AibH1 and AibH2, which is consistent with metal ion occupancy. The metal site density is clear more than 9 σ in 2*F*_o_ − *F*_c_ map and more than 8 σ in the metal omit *F*_o_ − *F*_c_ map. A metal content analysis indicated the presence of 1.96 Fe and 0.76 Zn per AibH1H2. The UV–Vis absorbance measurements of AibH1H2 showed no characteristic absorption (dashed line in Supplementary Fig. 7). On the other hand, when sodium azide was added to the enzyme, broad absorption was observed at 335, 350 and 460 nm (solid line in Supplementary Fig. 7). This emergent absorbance of azide-added AibH1H2 is similar to the absorbance properties of iron azide adducts observed in many other proteins, including diiron cores^26–29^. Moreover, the positions of the metal-coordinating amino acid residues in AibH2 are very similar to that in PtmU3—a non-heme diiron hydroxylase belonging to AHS^30^—thereby indicating that AibH2 also has a diiron catalytic center. In AibH2, the two Fe ions are coordinated by side-chains of three histidine and three carboxylate residues (Asp25^H2^, His27^H2^, His211^H2^, Glu265^H2^, Asp337^H2^, and His340^H2^), an ethylene glycol, and an oxygen atom (Fig. 5a). In the binuclear iron cluster containing oxygen bridges, the distances between Fe–O and Fe–Fe are 2.1/2.3 Å and 3.3 Å, respectively, and the Fe–O–Fe angle is 95°. These values suggest that AibH2 has a (μ-hydroxo)diiron complex^31^. AibH1 contains one Zn ion coordinated by side-chains of two histidine and three carboxylate residues (Asp24^H1^, His26^H1^, His201^H1^, Glu255^H1^, and Asp328^H1^) (Fig. 5b). Thus, the AibH1H2 heterotetrameric complex contains two catalytic AibH2 subunits, each with a diiron center, and two AibH1 subunits, each containing a zinc ion.

**Table 2:** Data collection and refinement statistics.

	AibH1H2 + GOL	AibH1H2 + EDO
*Data collection*		
Space group	*C*222_1_	*C*222_1_
*Cell dimensions*		
*a*, *b*, *c* (Å)	125.49, 208.05, 90.66	123.75, 206.68, 91.49
*α*, *β*, *γ* (°)	90.00, 90.00, 90.00	90.00, 90.00, 90.00
Resolution (Å)	38.710–2.70 (2.77–2.70)	45.92–2.45 (2.60–2.45)
*R*_sym_ or *R*_merge_	0.110 (0.356)	0.089 (0.471)
*I*/σ*I*	12.7 (3.6)	9.20 (1.98)
Completeness (%)	99.9 (99.2)	96.8 (98.9)
Redundancy	6.97 (7.15)	2.56 (2.64)
*Refinement*		
Resolution (Å)	45.12–2.75 (2.84–2.75)	45.92–2.45 (2.51–2.45)
No. reflections	31,229 (2767)	43,054 (2842)
*R*_work_/*R*_free_	0.190/0.250	0.184/0.236
*No. atoms*		
Protein	5572	5643
Ligand/ion	19	21
Water	96	124
*R.m.s deviations*		
Bond lengths (Å)	0.010	0.008
Bond angles (°)	1.417	0.986

One crystal was used for each data set. Values in parentheses are for highest-resolution shell.

### Identification of the enzymes in the cascade for D-MeSer catabolism

Some of the enzymes encoded in the *aib* gene cluster were strongly induced by Aib. By analyzing the activity of their recombinant proteins, their ability to catabolize D-MeSer and its products was discovered. In the Aib catabolic pathway in *R. wratislaviensis* C31-06, D-MeSer is oxidized to produce Amma—an α,α-dicarboxylic amino acid. The hydroxyl group of D-MeSer can undergo two-step dehydrogenation for conversion to a carboxy group by AibD1 and AibE. AibD1 is an NAD^+^-dependent d-MeSer dehydrogenase that oxidizes D-MeSer to 2-amino-2-methylmalonic semialdehyde (Amms), and AibE is an NAD^+^-dependent Amms dehydrogenase that oxidizes Amms into Amma (Fig. 2c). AibD1 has absolute stereospecific substrate specificity and reacted with D-MeSer but not with l-MeSer. AibD1 exhibited cofactor dependency for NAD^+^ with a specific activity of 18.1 μmol^−1^ min^−1^ mg^−1^ in the oxidation of D-MeSer. It did not use NADP^+^, which is consistent with the presence of the asparagine residue (Asp32) known to define cofactor specificity for NAD^+^ in the short-chain dehydrogenase/reductase superfamily^32^.

AibC is the first enzyme described to be an l-Ala-forming Amma decarboxylase. In a BLASTP search, AibC is annotated as a putative serine hydroxymethyltransferase, a PLP-dependent enzyme that catalyzes the transfer of a hydroxymethyl group between l-serine and tetrahydrofolate, forming glycine and 5,10-methylenetetrahydrofolate^33^. Some hydroxymethyltransferases are known to also act on l-MeSer to form d-Ala^34^. However, AibC had no hydroxymethyltransferase activity towards l-MeSer, d-MeSer, l-serine, or d-serine. Instead, AibC showed Amma decarboxylation activity with a specific activity of 18.7 μmol^−1^ min^−1^ mg^−1^. Two hydroxymethyltransferases, derived from rabbit liver cytosol and *Escherichia coli*, are reported to catalyze stereoselective decarboxylation of Amma to produce d-Ala^20^. In contrast to these previous observations, l-Ala was observed as the product of the AibC reaction (Fig. 2d). PLP is an essential cofactor for Amma decarboxylation by AibC, which explains why the addition of AOA induced the accumulation of Amma in *R. wratislaviensis* C31-06.

AibA is an NAD^+^-dependent l-Ala dehydrogenase with a specific activity of 768 μmol^−1^ min^−1^ mg^−1^, that acts in the final step of Aib catabolism to provide pyruvic acid for further *R. wratislaviensis* C31-06 metabolic pathways.

The elucidated Aib catabolic pathway in *R. wratislaviensis* C31-06 is summarized in Fig. 2a. Supplementary Table 1 shows the results of quantitative comparative proteome analysis of wild-type strain C31-06 and analysis of enzyme activity using each purified enzyme. Using the combination of protein amount and specific activity of each enzyme, the enzyme activities involved in Aib catabolism in the cells were calculated without endogenous background activities. The highest activity was observed in the last step, l-Ala dehydrogenation, which controls the direction of the catabolic metabolism.

### Construction of a d-MeSer-producing microorganism using the Aib monooxygenase

The recombinant *R. erythropolis* strain expressing AibH1H2GF was applied for the bioconversion of Aib to d-MeSer. d-MeSer has great industrial potential, for example, as a precursor of α-methyl-l-cysteine, which is an important synthetic intermediate of a selective inhibitor of inducible nitric oxide synthase^35^. However, only a small amount of d-MeSer was produced by this strain. Another *R. erythropolis* strain was constructed, expressing AibH1H2GF and AibT, and applied to the Aib to d-MeSer bioconversion. As shown in Table 1, AibT is the protein most highly induced by Aib and is a putative amino acid permease. It is encoded by *aibT*, which is adjacent to *aibF* in the *aib* gene cluster. Consequently, AibT functions as an amino acid permease transporting Aib and/or d-MeSer. The amount of d-MeSer produced increased substantially—reaching 32 mM—compared to the strain without AibT.

## Discussion

Although most members of the AHS are known to catalyze metal-dependent hydrolysis reactions, some also catalyze nonhydrolytic reactions, such as isomerization and decarboxylation. Recently, the AHS enzyme PtmU3 was reported as a β-hydroxylase in the biosynthesis of diterpenoids^30^; like AibH2, PtmU3 is a TIM-barrel fold protein with has a non-heme diiron center in the active site. AibH2 showed low amino acid similarity to the TIM barrel fold proteins similar to PtmU3. Furthermore, the substrate binding sites of AibH2 and PtmU3 were predicted by using CASTp^36^ (Supplementary Fig. 8). The largest pocket of each enzyme was located near the diiron center. The substrate-binding pocket of AibH2 showed less than half as much cavity as that of PtmU3 (412.3 Å^3^ vs. 1015.8 Å^3^, respectively). The difference in cavity size suggests that AibH2 can only accept substrates smaller than PtmU3, which accepts CoA-ligated substrates. Based on these results, AibH2 is an entirely different type of protein identified in this study. Although the amino acid sequence identity between AibH2 and *Streptomyces platensis* PtmU3 is only 25%, the residues needed to bind the Fe ions are highly conserved (Supplementary Fig. 9). However, there are some differences in metal coordination. The Glu313 of PtmU3 coordinates to an Fe ion, while the corresponding residue (Asp342) in AibH2 does not directly bind to, but indirectly interacts with, an Fe ion via a hydrogen bond network with water molecules replaced by ethylene glycol in the crystal structure. Although the His331 residue in AibH1 corresponding to His340 in AibH2 is conserved, the Asp residue corresponding to Asp342 in AibH2 is missing in the mononuclear AibH1. The acidic residue (Asp or Glu) at this position is conserved only in AHS enzymes with binuclear centers (Supplementary Fig. 10). So, the acidic residue is important for binding an additional metal ion to form a binuclear catalytic center in AHS enzymes. AibH1 is a monozinc protein and would be a noncatalytic subunit with an unknown, but essential, role in Aib hydroxylation. It may stabilize the heterotetrameric structure of AibH1H2, which enhances the binding affinity of the substrate. Alternatively, it may facilitate electron transport from ferredoxin to AibH2.

Some non-heme diiron enzymes form part of bacterial multicomponent monooxygenases (BMMs)^37^. BMMs are three-component or four-component enzyme systems that contain a hydroxylase complex, a regulatory protein, a ferredoxin:NAD^+^ oxidoreductase, and, in some cases, a Rieske-type ferredoxin. The soluble Rieske-type ferredoxin works in electron transfer between the FAD-containing oxidoreductase and the hydroxylase in some BMMs such as toluene-4-monooxygenase (T4MO)^38^. Here we found the electron transfer proteins AibF (an oxidoreductase) and AibG (a ferredoxin) in the Aib monooxygenase are similar to those in T4MO, except that AibF contains FMN instead of FAD. However, the hydroxylase complex of the Aib monooxygenase is composed of two AHS proteins with a TIM-barrel fold, unlike other BMMs in which the catalytic non-heme diiron enzymes in the hydroxylase complex usually have a 4-helix bundle fold. AibH1H2 hydroxylase is a completely different type of terminal oxygenase from other common hydroxylases in multicomponent monooxygenase systems, such as CYPs^24^ and Rieske dioxygenases (ROs)^39^. CYPs are monomeric heme-containing hydroxylases, and ROs consist of an α_3_ or α_3_β_3_ unit and their catalytic α-subunit contains a monoiron center and a Rieske-type [2Fe-2S] cluster. Unlike them, AibH1H2 is an α_2_α′_2_ heterotetrameric unit with a diiron center and does not have a heme molecule or an iron-sulfur cluster in its tertiary structure.

Selective hydroxylation of primary C–H bonds in mild conditions is still a challenging goal in industry. Soluble methane monooxygenase (sMMO)—a non-heme diiron enzyme—has the highest oxidation reactivity and can activate the very strong C–H bond in methane (105 kcal mol^−1^) as well as primary C–H bonds of other short-chain alkanes at ambient temperature and pressure^40,41^. Recombinant expression of multicomponent sMMO for practical use is still difficult with success only in methanotroph strains^42^. However, here, a complete set of the three-component Aib monooxygenase was easily expressed in an active form in recombinant *R. erythropolis* cells. Success in generating the d-MeSer-producing strain demonstrated that the Aib monooxygenase is a suitable biocatalyst for selective methyl group hydroxylation producing chiral hydroxylated compounds. Furthermore, we found several gene sets in other genomes having similar structure to the *R. wratislaviensis aibH1H2GF* gene set. The enzymes encoded are expected to form hydroxylation systems and may be valuable in industrial applications.

As demonstrated here, quantitative comparative proteome analysis is a powerful means to identify inducible proteins involved in catabolic pathways, especially multicomponent, or unstable enzymes and also nonenzyme proteins, such as transporters, which are usually difficult to identify by classical protein purification methods. The elucidated Aib catabolic pathway in *R. wratislaviensis* C31-06 includes several enzymes that catalyzes previously unreported reactions, and it will be of interest to understand how this microorganism evolved these enzymes to use Aib efficiently.

## Methods

### Enrichment cultivation and identification of Aib-degrading microorganisms

Microorganisms from natural sources in Japan were cultivated at 28 °C in the enrichment medium comprised 0.1% (w/v) Aib, 0.1% (w/v) NH_4_Cl, 0.1% (w/v) KH_2_PO_4_, 0.1% (w/v) K_2_HPO_4_, 0.03% (w/v) MgSO_4_·7H_2_O, and 0.01% (w/v) yeast nitrogen base w/o amino acids and ammonium sulfate (BD Difco, MD, USA). After several inoculations into fresh medium, microorganisms in the culture broth were isolated on 1.5% (w/v) agar plates of the medium. Genomic DNA was extracted from microbial cells using a DNeasy Blood & Tissue Kit (Qiagen, Hilden, Germany). The 16S ribosomal DNA gene was amplified by PCR using the following primers, 5′-GTGCCAGCMGCCGCGG-3′ and 5′-GGTTACCTTGTTACGACTT-3′, and sequenced.

### Analytical methods used for the catabolites derived from Aib

The LC-MS analysis for amino acids was carried out on an LCMS-2010A system (Shimadzu, Kyoto, Japan) equipped with an XBridge C18 column (4.6 × 150 mm; Waters, MA, USA) heated to 40 °C. The mobile phase was 10 mM ammonium acetate (pH 5.0, eluent A) and methanol (eluent B), at a flow rate of 0.30 mL min^−1^. The eluent gradient was 0% (v/v) eluent B for 0–0.1 min, 0–1% B for 0.1–0.5 min, 1–5% B for 0.5–18 min, 5–9% B for 18–19 min, 9–17% B for 19–29.5 min, and 17–60% B for 29.5–40 min. Amino acids were derivatized by AccQ-Tag fluorescence derivatization reagent (Waters) according to the manufacturer’s instructions. The amino acid derivatives were determined with fluorescence detection at excitation wavelength 250 nm and emission wavelength 395 nm and with electrospray ionization-mass spectrometry (ESI-MS). The chiral HPLC analysis of amino acids was performed using an LCMS-2010A system equipped with a COSMOSIL 5C_18_-AR-II column (4.6 × 150 mm; Nacalai Tesque, Kyoto, Japan) heated to 40 °C. The mobile phase was 10 mM phosphate buffer (pH 3.0) and methanol (65/35, v/v), and the flow rate was 1.0 mL min^−1^. Amino acids were dissolved in 50 μL 0.40% (v/v) triethylamine in acetonitrile, and then derivatized with 50 μL 0.2% (w/v) 2,3,4,6-tetra-*O*-acetyl-β-d-glucopyranosyl isocyanate (GITC) in acetonitrile at room temperature for 30 min. The amino acid derivatives were monitored by UV absorption at 254 nm and ESI-MS. Acetone was measured by salicylaldehyde method^43^. In brief, 40 μL samples were mixed with 2 μL salicylaldehyde and 30 μL 19.6 M potassium hydroxide and then incubated at room temperature for 20 min. The mixture was diluted with 128 μL distilled water and acetone concentration was determined by measuring UV absorbance at 474 nm.

### Biodegradation of Aib in the resting cell reaction

Soil-isolated microorganisms were grown and harvested by centrifugation at 8000 × *g* for 10 min, washed twice with 0.85% (w/v) NaCl, and used for the resting cell reaction. A mixture consisting of 10 mM Aib, 10 mM glucose, and 5% (w/v) wet cells in 50 mM HEPES buffer (pH 7.5) was incubated at 28 °C with shaking at 300 rpm for 4 h. To inhibit PLP-dependent enzyme activity, 1 mM AOA was added to the reaction mixture. The reaction mixture was centrifuged at 7000 × *g* for 10 min at 4 °C, and the supernatant was used for amino acid and acetone analyses.

### Purification of 2-amino-2-methylmalonic acid (Amma)

*R. wratislaviensis* C31-06 was grown in the enrichment medium supplemented with 0.05% (w/v) glucose until the optical density at 600 nm (O.D._600_) reached 1.0. The cells were then collected by centrifugation and washed twice with 0.85% (w/v) NaCl. The wet cells (about 1.2 g) were suspended in 50 mL of a reaction mixture comprising 10 mM D-MeSer, 20 mM AOA, and 50 mM Tris-HCl (pH 7.5). The resting cell reaction was carried out at 28 °C for 5 days with shaking at 120 rpm. The reaction mixture was centrifuged, and the supernatant was applied to Dowex 1 × 8 anion exchange resin (Dow Chemical Co., MI, USA). After washing with 100 mL of distilled water, Amma was eluted with 100 mL 4.0 M acetic acid. The fractions containing Amma were collected and applied to Dowex 50 W × 8 cation exchange resin (Dow Chemical Co.). Amma was eluted with distilled water and the fractions containing Amma were collected. Amma was freeze-dried, dissolved in D_2_O, and analyzed by NMR spectroscopy and mass spectrometry. The ^1^H-NMR and ^13^C-NMR spectra were recorded on an Avance 500 (Bruker, MA, USA). The spectra of Amma were as follows: ^1^H NMR (500 MHz, D_2_O) *δ*: 1.71 (s, 3H), ^13^C NMR (125 MHz, D_2_O) *δ*: 19.79, 63.92, 171.04. ESI-MS (*m*/*z*) = 134 [M+H]^+^.

### Draft genome sequence of *R. wratislaviensis* C31-06

*R. wratislaviensis* C31-06 genomic DNA was prepared and sequenced by Hokkaido System Science Co. (Hokkaido, Japan) using the Illumina MiSeq system (Illumina, CA, USA). The genome sequence was assembled using CLC Genomics Workbench 7.5 (CLC bio, Aarhus, Denmark) and functionally annotated using Rapid Annotation on the Subsystem Technology (RAST) server. The genome totaled 9,431,679 base pairs, consisting of 114 contigs with a G+C content of 67.2% and 9016 open reading frames (ORFs).

### Quantitative comparative proteome analysis by LC-MS/MS

*R. wratislaviensis* C31-06 cells were prepared in Aib-inducible or non-inducible conditions. The enrichment medium with 0.05% (w/v) glucose was used for induction, and medium without Aib was used for non-induction. The addition of 0.05% glucose helped the growth of C31-06 cells without inhibiting the Aib-degrading activity. *R. wratislaviensis* C31-06 was cultivated in each medium for 38.5 h. Sample preparation and proteome analysis were performed by previously reported with slight modification^21^. Cells were disrupted with 0.10 mm beads using a multibeads shocker (Yasui Kikai, Osaka, Japan), and digested with trypsin. The six tryptic digests, including triplicate Aib-induction and non-induction samples, were labeled using a tandem mass tag (TMT) 6-plex labeling kit (Thermo Fisher Scientific, MA, USA), and then dissolved in 60 μL 0.1% (v/v) formic acid. LC-MS/MS was conducted on an Ultimate 3000 HPLC and an LTQ Orbitrap Velos Mass Spectrometer (Thermo Fisher Scientific) with a monolithic silica capillary column (500 cm long, 0.1 mm ID)^44^.

### Bioconversion of Aib into d-MeSer in recombinant strains heterologously expressing the Aib monooxygenase

Gene fragments of *aibH1H2GF*, *aibH1H2G*, *aibH1H2F*, *aibH1GF*, *aibH2GF*, and *aibT* were amplified by PCR with the primers shown in Supplementary Table 2. The DNA fragments were cloned into pTipQC1 or pTipRT2 (Hokkaido System Science Co.) previously digested with *Nco*I and *Hin*dIII or *Nde*I and *Hin*dIII, using NEBuilder HiFi DNA Assembly (New England Biolabs, MA, USA). The resultant plasmids were transformed into *R. erythropolis* L88 (Hokkaido System Science Co.). The recombinant strains were cultured at 28 °C in LB medium containing 20 µg mL^−1^ chloramphenicol, and 5 µg mL^−1^ tetracycline if necessary. The culture was supplemented with 0.2 µg mL^−1^ thiostrepton and 20 mM Aib at O.D._600_ of 0.8 and was shaken at 28 °C for 16 h. The broth was centrifuged at 7000 × *g* for 10 min at 4 °C, and the supernatant was used for the amino acid analysis.

### Preparation of the recombinant enzymes encoded by the *aib* gene cluster

Each gene encoded in the *aib* gene cluster of *R. wratislaviensis* C31-06 was amplified by PCR using primers shown in Supplementary Table 2. The DNA fragments were cloned into pQE-80L (Qiagen) or pTipQC1 previously digested with *Bam*HI and *Hin*dIII or *Nco*I and *Hin*dIII, using NEBuilder HiFi DNA Assembly Master Mix. The resulting plasmids were transformed into *R. erythropolis* L88 or *E. coli* JM109. The recombinant *R. erythropolis* strains were cultured at 28 °C in LB medium containing 20 µg mL^−1^ chloramphenicol. The culture was supplemented with 0.2 µg mL^−1^ thiostrepton at O.D._600_ of 0.8 and was shaken at 28 °C for 16 h. The recombinant *E. coli* strains were cultivated at 28 °C in LB medium supplemented with 50 μg/mL ampicillin. After O.D._600_ was reached to 0.8, 1.0 mM isopropyl-β-d-thiogalactopyranoside (IPTG) was added for protein expression and incubation continued at 20 °C for 16 h. Cells were harvested and washed twice with 0.85% NaCl and resuspended in 20 mM Tris-HCl buffer (pH 8.0) containing 0.50 M NaCl and 20 mM imidazole. The *R. erythropolis* cells were disrupted by glass beads using a Multi-Beads Shocker, and *E. coli* cells were disrupted by ultrasonication using Insonator 201R (Kubota, Tokyo, Japan) and centrifuged. The enzymes were purified from the supernatant by using a HisTrap HP column, a Mono Q 5/50 column, and a Superdex 200 Increase 10/300 GL column (GE healthcare). The purified enzymes were ultrafiltered and the buffer was exchanged with 20 mM Tris-HCl buffer (pH 8.0).

### Analytical assays of AibF

The diaphorase activity of AibF was assayed using 2,6-dichlorophenolindophenol (DCPIP) as a two-electron acceptor. The reaction mixture comprised 400 µM NAD(P)H, 100 µM DCPIP, and 0.76 µg mL^−1^ AibF in 100 mM potassium phosphate buffer (pH 7.4), and was incubated at 20 °C. The DCPIP reduction activity was spectrophotometrically determined with a molar extinction coefficient of 21.0 mM^−1^ cm^−1^ at 600 nm^45^. For the flavin cofactor analysis of AibF, noncovalently bound cofactors were released from AibF by heating the enzyme solution (5.4 mg mL^−1^) to 99 °C for 5 min in the dark. Subsequently, the solution was incubated on ice for 2 min and centrifuged at 18,000 × *g* for 15 min. The supernatant was used for HPLC analysis, which was performed on a Shimadzu LC-VP system equipped with an Atlantis T3 column (5 µm, 4.6 × 250 mm; Waters). The mobile phase was 10 mM K_2_HPO_4_ (pH 6.0, eluent A) and methanol (eluent B), and the flow rate was 1.0 mL min^−1^. The eluent gradient was 15% (v/v) eluent B for 0–5 min, 15–65% B for 5–20 min, 65–100% B for 20–25 min, 100% B for 25–35 min. The flavin cofactors were detected with the fluorescence detector at excitation wavelength 450 nm and emission wavelength 520 nm.

### Activity assays for d-MeSer dehydrogenase (AibD1), and Amms dehydrogenase (AibE)

To determine D-MeSer dehydrogenase activity of AibD1, reaction mixtures containing 3.0 mM substrate, 2.0 mM NAD(P)^+^, 30 μg mL^−1^ enzyme in 50 mM Tris-HCl buffer (pH 9.0) were incubated at 37 °C. The enzyme activities were determined by measuring the UV absorption of NAD(P)H at 340 nm. The amount of NAD(P)H was determined using a molar extinction coefficient of 6.22 mM^−1^ cm^−1^. For the coupling reaction of AibD1 and AibE, reaction mixtures containing 3.0 mM substrate, 2.0 mM NAD(P)^+^, 30 μg mL^−1^ AibD1, 30 μg mL^−1^ AibE, in 50 mM Tris-HCl buffer (pH 9.0) were incubated at 37 °C for 3 h, and the produced Amma was analyzed.

### Activity assays for Amma decarboxylase (AibC)

For the decarboxylase assay, the reaction mixture consisted of 20 mM Amma, 0.1 mM PLP, and 3.2 μg mL^−1^ AibC in 100 mM HEPES buffer (pH 8.0) and was incubated at 28 °C for 30 min. The Amma decarboxylation activity of AibC was determined by the amount of l-Ala formed in the amino acid analysis. For the hydroxymethyltransferase assay, the reaction mixture comprised 20 mM substrate, 0.1 mM PLP, 0.5 mM tetrahydrofolic acid, and 3.2 μg mL^−1^ AibC in 100 mM HEPES buffer (pH 8.0) and was incubated at 28 °C for 30 min. The hydroxymethyltransferase activity of AibC was determined by the amount of product in the amino acid analysis.

### Activity assay for l-Ala dehydrogenase (AibA)

The reaction mixture contained 5.0 mM l-Ala, 2.0 mM NAD(P)^+^, and 14 μg mL^−1^ AibA in 50 mM CAPS buffer (pH 10.0) and was incubated at 30 °C for 30 min. Enzyme activities were determined by measuring the UV absorption of NAD(P)H at 340 nm. The amount of NAD(P)H was determined using a molar extinction coefficient of 6.22 mM^−1^ cm^−1^.

### Crystallization and X-ray structure determination of AibH1H2 complex

Selenomethionine (SeMet)-derivatized AibH1H2 was expressed in *E. coli* JM109 by Overnight Express Autoinduction System 2 (Merck KGaA, Darmstadt, Germany) according to the manufacturer protocol. The enzyme was purified with HisTrap HP column in the same way as described above. The purified enzyme was confirmed to be homogeneous using SDS-PAGE and then concentrated to 4.5 mg mL^−1^ by ultrafiltration with Amicon Ultra (30 KDa cut-off; Merck KGaA). The protein solution for crystallization consists of 20 mM Tris-HCl (pH 7.8), 10 mM NaCl and 1 mM DTT. The SeMet-derivatized AibH1H2 was crystallized at 293 K using sitting-drop vapor diffusion. Single crystals were obtained at 20 °C in a mixture of protein solution (1 μL) and mother liquor (1 μL) [0.1 M Tris-HCl (pH 8.5), 1 M ammonium sulfate and 12% (v/v) glycerol]. The crystal was mounted on a nylon loop (Hampton Research, CA, USA) after cryoprotection in crystallization solution supplemented with 30% glycerol (GOL) or 25% ethylene glycol (EDO) and placed directly or flash-cooled in a cold nitrogen-gas stream at 100 K. AibH1H2 crystallizes in the *C*222_1_ space group with two molecules in the asymmetric unit. Diffraction data were collected at a *λ* of 0.9791 Å using a Dectris Pilatus3 S6M detector at the BL5A station of the Photon Factory in KEK (Tsukuba, Japan) and at a *λ* of 1.00 Å using a Dectris EIGER4M detector at the BL26B1 station of SPring-8 (Hyogo, Japan). Diffraction data were indexed, integrated, and scaled with the XDS program^46^. The crystal structure of AibH1H2 was solved by the single-wavelength anomalous diffraction method using the SeMet AibH1H2 crystal. Selenium sites, initial phasing, and density modifications were done using the program SHELX^47^. The Coot program^48^ was used for the modification of the initial model. Phases were computed to 2.8 Å resolution with the PHENIX Autosol program^49^. The subsequent structure was solved by molecular replacement using the Molrep program in the CCP4 program package^50^ with the above model as the search model. Refinement and model building steps were performed with the programs Refmac5^51^, PHENIX Refine^49^, and COOT. A summary of the processing and refinement statistics for all structures can be found in Table 2. Ribbon plots were prepared using the PyMOL program^52^.

### Metal content analysis

The purified AibH1H2 was ultrafiltered and the buffer was exchanged with the water for ultratrace analysis. An aliquot 100 μL of 13.2 mg/mL AibH1H2 was mixed with 1 mL of 5% v/v nitric acid and subsequently diluted with ICP analysis grade water up to 5 mL. The sample was quantified by inductively coupled plasma mass spectrometry (ICP-MS; Agilent 7500cx, Agilent Technologies, CA, USA). Calibration was carried out by authentic element solution according to manufacturer’s instruction.

### Statistics and reproducibility

All cell culture and enzyme experiments were repeated three times and are presented as a mean ± standard deviation. Analysis of the triplicate proteome data was performed using Proteome Discoverer 1.4 (Thermo Fisher Scientific). Protein identification was performed using the Mascot algorithm against the *R. wratislaviensis* C31-06 protein database (9016 sequences), with a precursor mass tolerance of 20 ppm and a fragment ion mass tolerance of 50 mmu in quantitative proteome analysis, and a mass tolerance of 2.0 Da and a fragment ion mass tolerance of 0.8 Da in qualitative proteome analysis. Protein quantification was performed using the reporter ions quantifier with the TMT 6-plex method. The data were then filtered with cut-off criteria of *q* ≤ 0.05, corresponding to a 5% false discovery rate on a spectral level. In the qualitative proteome analysis, proteins with scores >10 were accepted as identified proteins. Global median normalization was performed to normalize the quantity of each tryptic digest injected into the mass spectrometer.

### Reporting summary

Further information on research design is available in the Nature Research Reporting Summary linked to this article.

## Supplementary information

Supplementary Information

Description of Additional Supplementary Files

Supplementary Data 1

Reporting Summary

## Data Availability

Sequence data and assemblies have been deposited in GenBank under BioProject PRJDB7624. The datasets generated and/or analyzed during the current study are available in the jPOST repository (JPST001014, PXD022656)^53^. Atomic coordinates and structure factors have been deposited in the Protein Data Bank under accession codes 6M1W (AibH1H2 + GOL) and 6M2I (AibH1H2 + EOD).
