# Supplementary material for: A three-component monooxygenase from Rhodococcus wratislaviensis may expand industrial applications of bacterial enzymes
Source: Commun Biol. 2021 Jan 4;4:16. doi: 10.1038/s42003-020-01555-3 (PMC7782822; doi:10.1038/s42003-020-01555-3)
Supplement: Supplementary file 1 — Supplementary Information [file 42003_2020_1555_MOESM1_ESM.pdf]

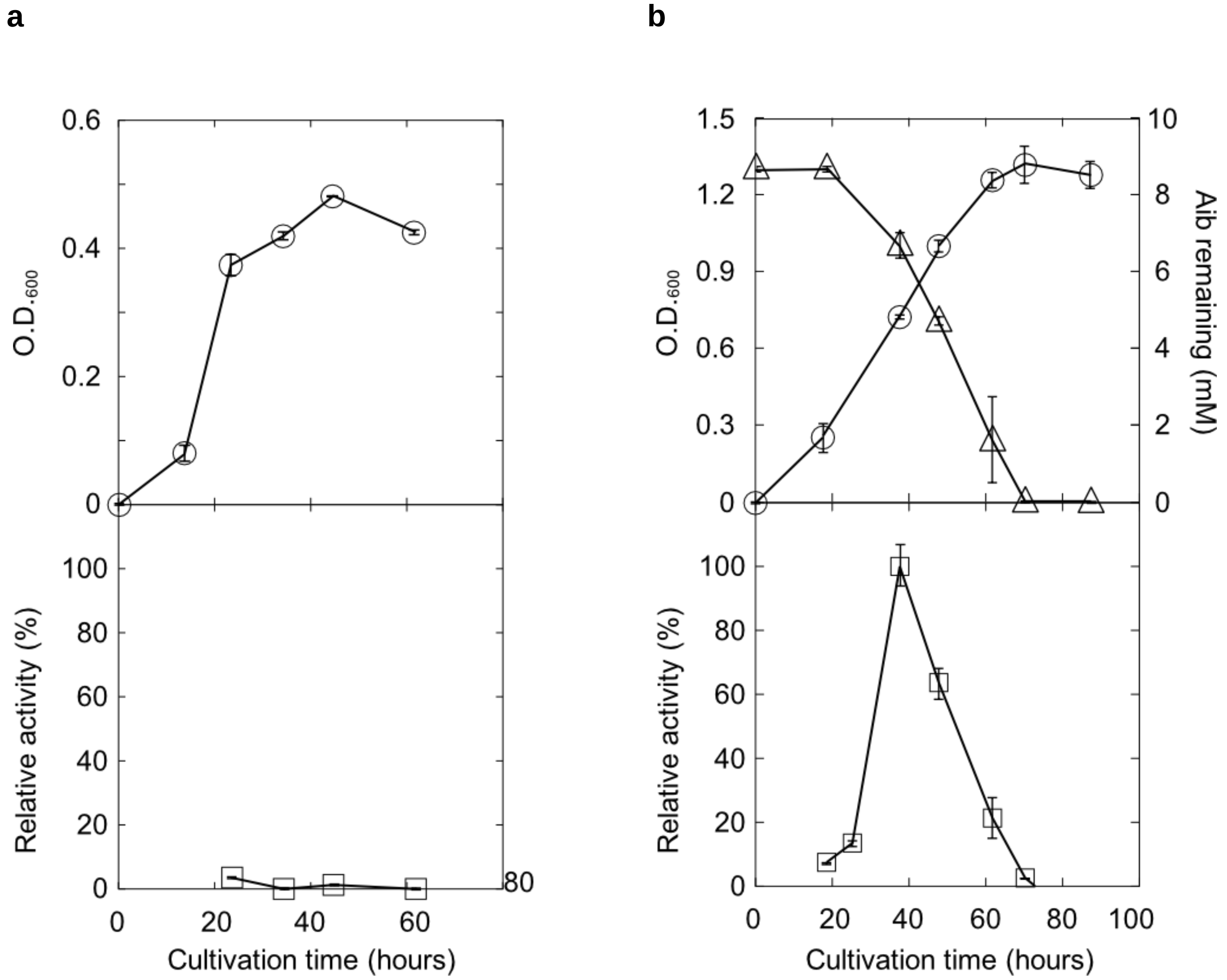

Supplementary Figure 1: Conversion of Aib into D-MeSer in *R. wratislaviensis* C31-06. Cells were harvested at various time points from the culture medium without **a** Aib or **b** with 0.1% (w/v) Aib and used for the bioconversion of Aib. Open circles, O.D.$_{600}$; open triangles, Aib in the culture medium; open squares, relative activity for Aib conversion. Data represent the mean (±SD) of n=3 independent experiments. Error bars denote SD.

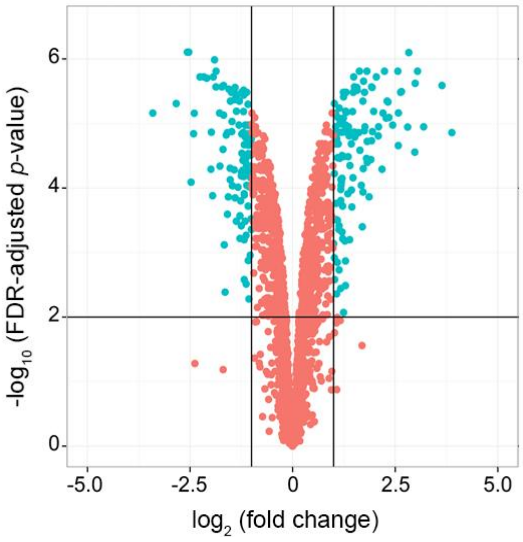

Supplementary Figure 2: Volcano plot of the quantitative comparative proteome analysis data. The data for all proteins are plotted as $\log_2$ (fold change of Aib-induction, logFC) versus the $-\log_{10}$ (FDR-adjusted *p*-value). Thresholds (fold change > 2.0, *p*-value < 0.01) are shown as solid lines. Proteins selected as significantly different are highlighted as green dots.

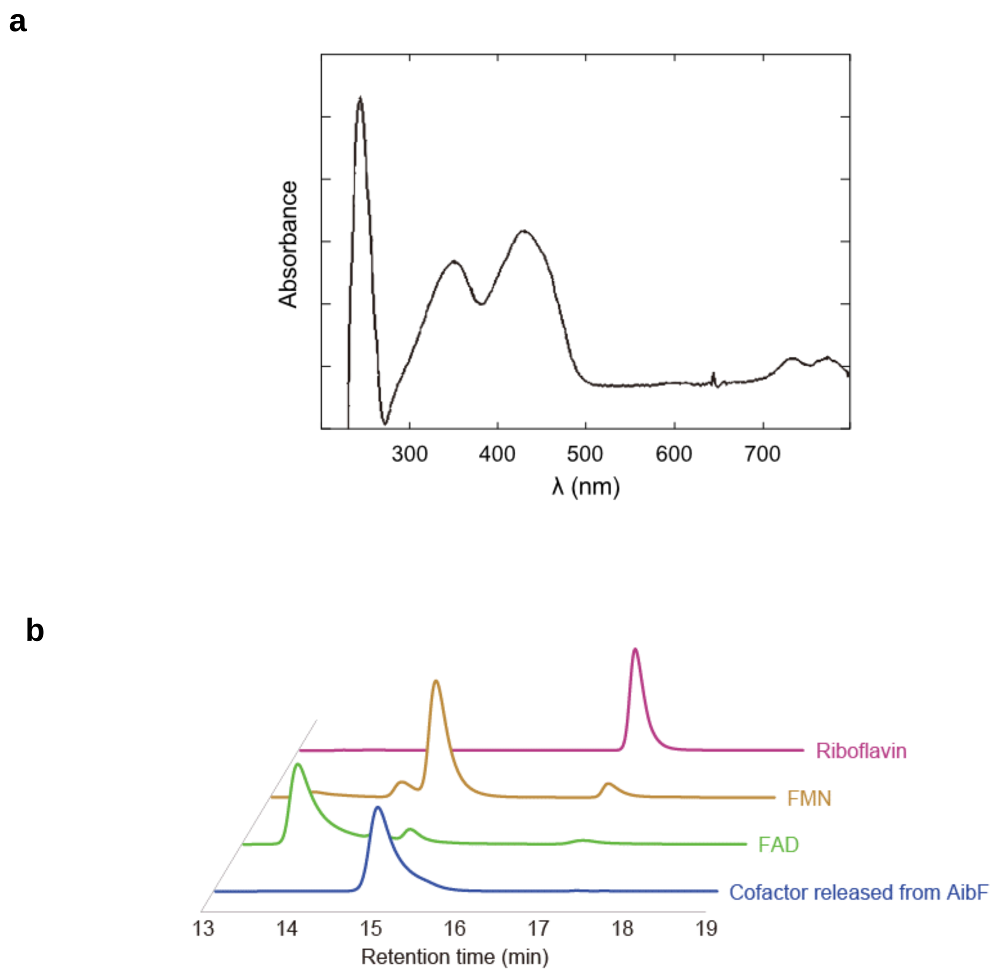

Supplementary Figure 3: Identification of AibF cofactor. The supernatant from the heat-treated AibF solution was analyzed by **a** spectral analysis and **b** cofactor analysis using HPLC. UV-VIS absorption spectrum was collected under non-reducing conditions and AibF concentration was 5.4 mg mL^{-1}. Major peaks were detected at 370 nm and 446 nm. In HPLC analysis, standard reagents, riboflavin, FMN, and FAD, were also analyzed.

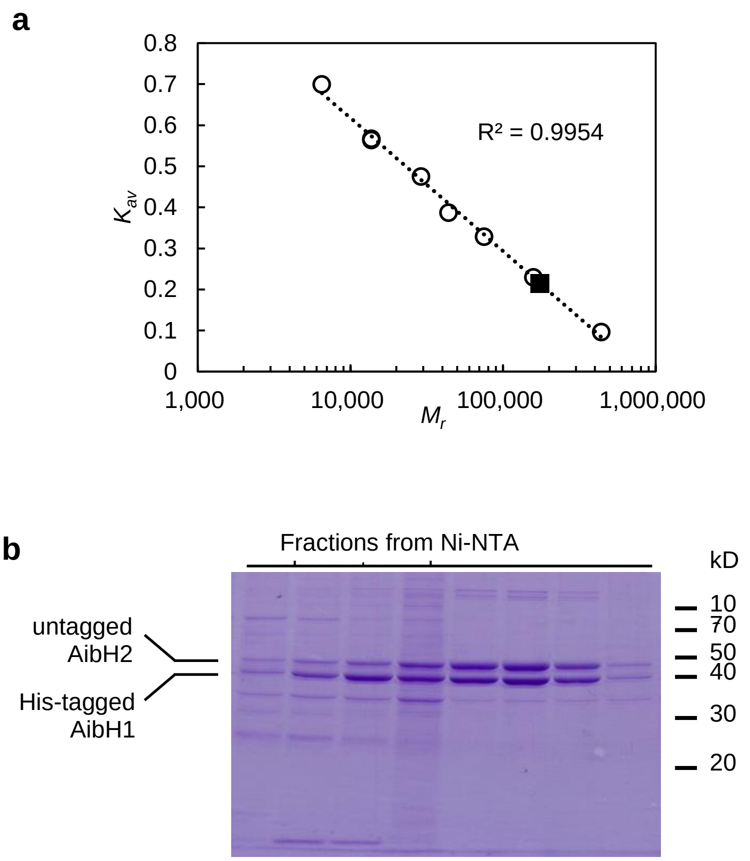

Supplementary Figure 4: Analysis of the Aib hydroxylase protein complex, AibH1H2. The AibH1H2 complex was characterized by **a** gel filtration analysis and **b** SDS-PAGE analysis. Relative molecular mass standards (open circles) used in the gel filtration analysis were as follows: Aprotinin (6,500), Ribonuclease A (13,700), Carbonic anhydrase (29,000), Ovalbumin (44,000), Conalbumin (75,000), Aldolase (158,000), and Ferritin (44,000). The AibH1H2 complex is shown by a filled square. The relative molecular mass of the AibH1H2 complex to be approximately 180,000. The elution fractions from Ni-NTA chromatography were separated by SDS-PAGE. The relative molecular mass of AibH1 and AibH2 are 43,000 and 42,000, respectively.

a

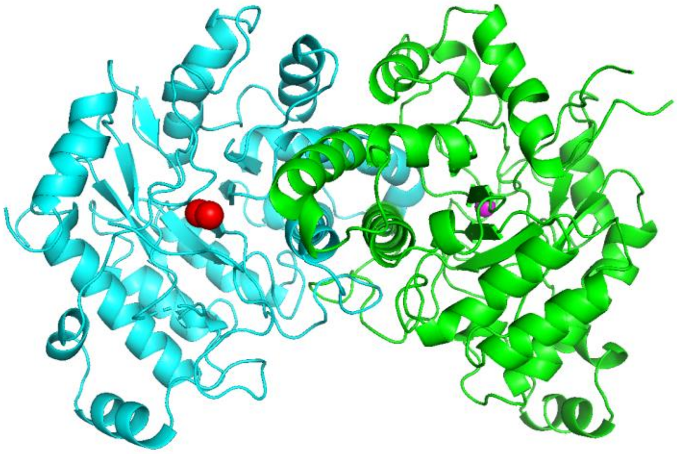

b

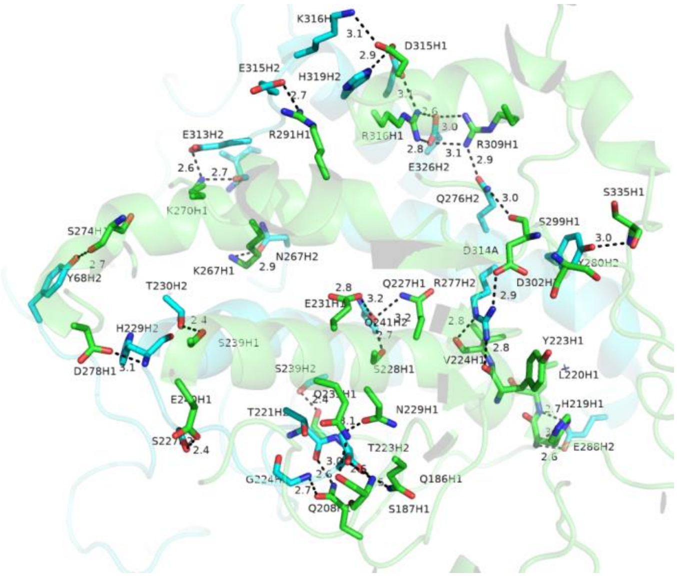

Supplementary Figure 5: Dimer assembly of AibH1 and AibH2. **a** Ribbon representation of the AibH1 subunit (green) and the AibH2 subunit (cyan) in an asymmetric unit. **b** The interaction between AibH1 and AibH2. AibH1 residues (the green sticks), AibH2 residues (the cyan sticks), and hydrogen bonds (the black dashes) are shown.

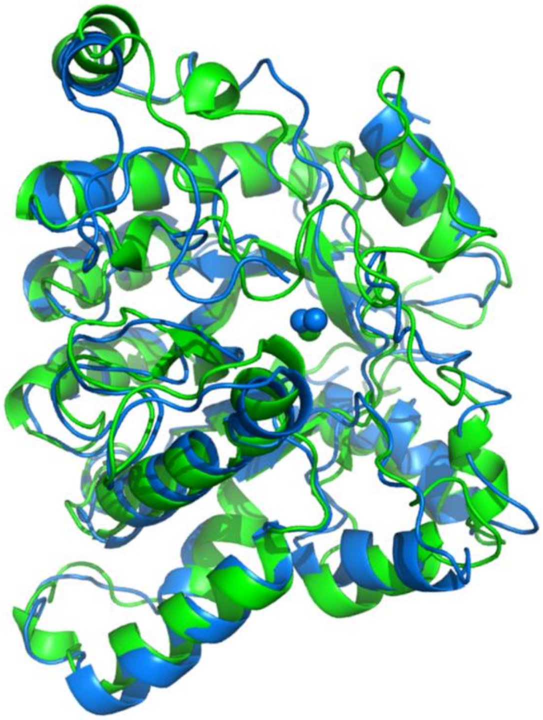

Supplementary Figure 6: Structural superposition of AibH1 and AibH2 subunits. The ribbon diagram of AibH1 subunit (green) and AibH2 subunit (blue) are shown.

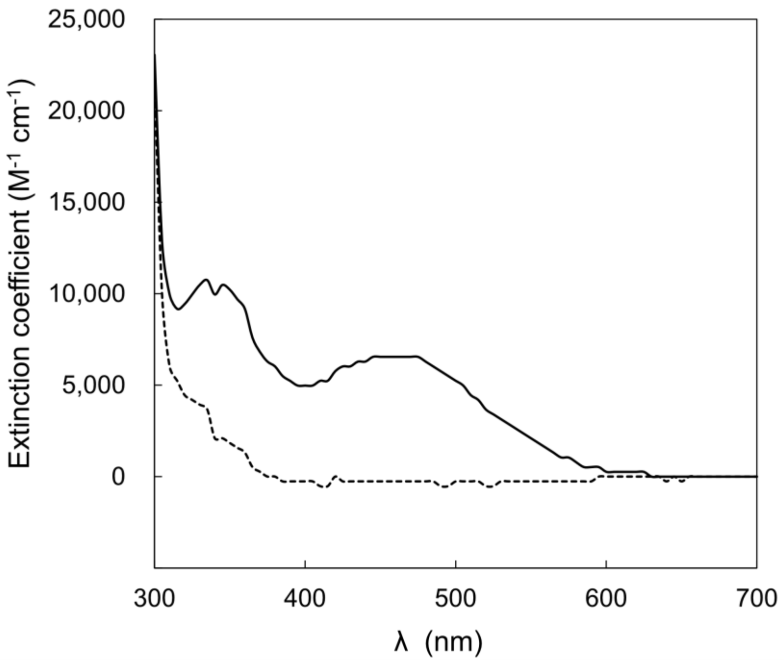

Supplementary Figure 7: UV-Visible absorption spectra of AibH1H2. The spectra of AibH1H2 isolated (dashed line) and reacted with 2 M sodium azide (solid line). AibH1H2 (326 μg mL^{-1}) in 10 mM HEPES (pH 7.8) buffer was used.

a

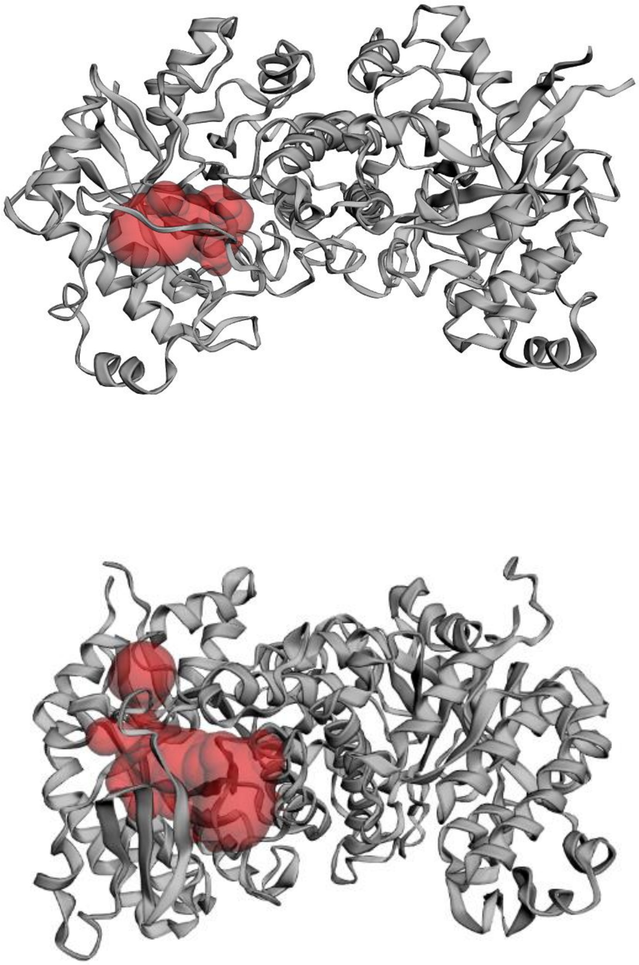

b

Supplementary Figure 8: Substrate binding sites prediction. Cavities in **a** AibH2 and **b** PtmU3 were calculated by CASTp program. The largest pockets by volume identified was shown in red and the ribbon diagram of proteins are shown in grey. Pocket volumes (Å^3) were calculated using the default probe sphere radius of 1.4 Å.

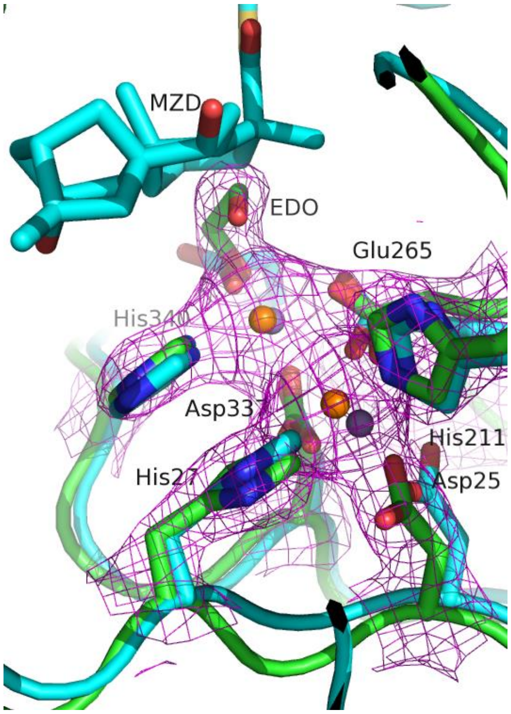

Supplementary Figure 9: Comparison of the catalytic centers between AibH2 and PtmU3. In AibH2, the amino acid residues involved in metal coordination and EDO are shown as green sticks, and two Fe ions are shown as orange spheres. In PtmU3 (PDB ID: 6OMQ), the amino acid residues involved in metal coordination and MZD are shown as cyan sticks, and two Fe ions are shown as purple spheres. The ($2F_o$ - F_c) OMIT electron-density map (magenta mesh) contoured at 1.5 σ is superimposed.

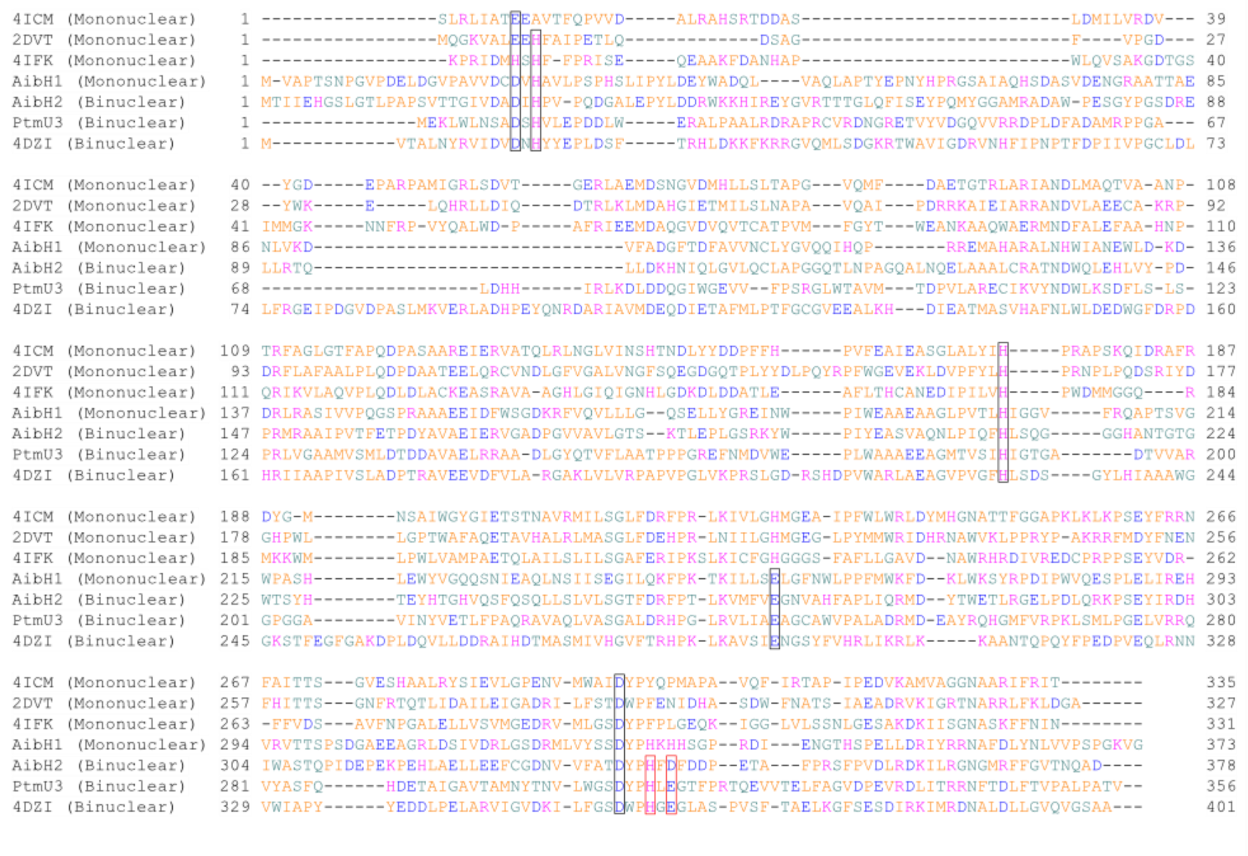

Supplementary Figure 10: Sequence alignment of AibH1, AibH2, PtmU3, and selected AHS enzymes in the PDB. The residues to bind metal ions are enclosed in frames, and the two important residues in the formation of binuclear sites are highlighted with red frames. AibH2, 2DVT, 4ICM, and 4IFK have a mononuclear metal center, and AibH2, PtmU3, and 4DZI have a binuclear metal center. PDB entries: 2DVT, Zn-dependent 2,6-dihydroxybenzoate decarboxylase from *Rhizobium* sp.; 4ICM, 5-carboxyvanillate decarboxylase LigW from *Sphingomonas paucimobilis*; 4IFK, α-amino-β-carboxymuconate-ε-semialdehyde decarboxylase from *Pseudomonas fluorescens*; 4DZI, amidohydrolase from *Mycobacterium avium*.

Supplementary Table 1 Specific activities of recombinant proteins relating to Aib catabolism.

Protein	logFC[a]	Specific activity			
		μmol^{-1} min^{-1} mg^{-1} of purified protein	Assayed reaction	nmol^{-1} min^{-1} ml^{-1} of culture	Assayed reaction
AibH1	2.24	-	-	8.33±0.85[d]	Aib hydroxylation
AibH2	2.57	-	-		
AibG	2.09	-	-		
AibF	2.18	271 ± 13[b]	DCPIP reduction		
AibD1	2.35	18.1±1.9[c]	D-MeSer dehydrogenation	-	-
AibE	2.31	*n.d.*	*n.d.*	-	-
AibC	2.20	18.7±1.4[d]	Amma decarboxylation	-	-
AibA	2.32	768±16[e]	L-Ala dehydrogenation	-	-

a, log$_2$ (fold change of Aib-induction) in the quantitative comparative proteome analysis. *b*, the amount of reduced DCPIP produced was determined. *c*, the amount of NADH produced was determined. *d*, the amount of L-Ala produced was determined. *e*, the amount of D-MeSer produced was determined in the *R. erythropolis* L88 culture expressing AibH1H2GF and AibT. *n.d.*, not determined. Data are representative of n=3 independent experiments and values are expressed in mean (±SD).

Supplementary Table 2 Primers used in this study.

Target gene	Primer	Sequence (5′-3′)	Description
aibF	aibF-F1	TCGCATCACCATCACCATCACGGATCCATGACCAATTCAGATAGTTC	Construction of AibF expression plasmid for *E. coli*
	aibF-R1	CAACAGGAGTCCAAGCTCAGCTAATTACTAGAGATCGAGGACGAGCC	
aibH1H2	aibH1H2-F1	TCGCATCACCATCACCATCACGGATCCATGGTTGCACCAACCTCGAA	Construction of AibH1H2 co-expression plasmid for *E. coli*
	aibH1H2-R1	CAACAGGAGTCCAAGCTCAGCTAATTATTAGTCCGCCTGATTCGTAA	
aibH1H2GF	aibH1H2GF-F	TGTTTAACTTTAAGAAGGAGATATACCATGGTTGCACCAACCTCGAA	Construction of AibH1H2GF co-expression plasmid for *R. erythropolis*
	aibH1H2GF-R	TGGTGATGGTGATGCTCGAGAGATCTACTAGAGATCGAGGACGAGCC	
aibT	aibT-F	GTTTAACTTTAAGAAGGAGATATACATATGACAGTGAATCATTCCCA	Construction of AibT expression plasmid for *R. erythropolis*
	aibT-R	TGGTGATGGTGATGCTCGAGAGATCTATCAGATTCTGGGCTGCAGAA	
aibH1H2G	aibH1H2G-F	TGTTTAACTTTAAGAAGGAGATATACCATGGTTGCACCAACCTCGAA	Construction of AibH1H2G co-expression plasmid for *R. erythropolis*
	aibH1H2G-R	TGGTGATGGTGATGCTCGAGAGATCTATCAGACCGTCAGGTCCGTTT	
aibH1H2F	aibH1H2-F2	TGTTTAACTTTAAGAAGGAGATATACCATGGTTGCACCAACCTCGAA	Construction of AibH1H2F co-expression plasmid for *R. erythropolis*
	aibH1H2-R2	CGTCCGAACTATCTGAATTGGTCATTCTTAGTCCGCCTGATTCGTAA	
	aibF-F2	CGGCGTTACGAATCAGGCGGACTAAGAATGACCAATTCAGATAGTTC	
	aibF-R2	TGGTGATGGTGATGCTCGAGAGATCTACTAGAGATCGAGGACGAGCC	
aibH1GF	aibH1-F	TGTTTAACTTTAAGAAGGAGATATACCATGGTTGCACCAACCTCGAA	Construction of AibH1GF co-expression plasmid for *R. erythropolis*
	aibH1-R	CGCTACCGATTACAAACTTGGACATTCTTAACCAACCTTTCCTGGGC	
	aibGF-F	CCCGAGCCCAGGAAAGGTTGGTTAAGAATGTCCAAGTTTGTAATCGG	
	aibGF-R	TGGTGATGGTGATGCTCGAGAGATCTACTAGAGATCGAGGACGAGCC	
aibH2GF	aibH2GF-F	TGTTTAACTTTAAGAAGGAGATATACCATGACCATCATCGAACACGG	Construction of AibH2GF co-expression plasmid for *R. erythropolis*
	aibH2GF-R	TGGTGATGGTGATGCTCGAGAGATCTACTAGAGATCGAGGACGAGCC	
aibA	aibA-F1	TCGCATCACCATCACCATCACGGATCCATGAGGATCGGTATCCCCCG	Construction of AibA expression plasmid for *E. coli*
	aibA-R1	CAACAGGAGTCCAAGCTCAGCTAATTATCACCAGCTAGCGGCGGTGT	
aibC	aibC-F1	ATGGGCCATCACCATCACCATCACGCCATGGATCACTCGACCATCGC	Construction of AibC expression plasmid for *R. erythropolis*
	aibC-R1	TGGTGATGGTGATGCTCGAGAGATCTATTAGACCGAGCTCCCGTCCT	
aibD1	aibD1-F	TCGCATCACCATCACCATCACGGATCCATGACCAGCACTATTGGGTT	Construction of AibD1 expression plasmid for *E. coli*
	aibD1-R	CAACAGGAGTCCAAGCTCAGCTAATTACTATTCTGGAATCTGCTCAT	
aibE	aibE-F	TCGCATCACCATCACCATCACGGATCCATGGCGATTCTCGATGCTAC	Construction of AibE expression plasmid for *E. coli*
	aibE-R	CAACAGGAGTCCAAGCTCAGCTAATTAGTGACTGATATCGATCG	
