# Supplementary material for: A three-component monooxygenase from Rhodococcus wratislaviensis may expand industrial applications of bacterial enzymes
Source: Commun Biol. 2021 Jan 4;4:16. doi: 10.1038/s42003-020-01555-3 (PMC7782822; doi:10.1038/s42003-020-01555-3)
Supplement: Supplementary file 2 — Description of Additional Supplementary Files [file 42003_2020_1555_MOESM2_ESM.pdf]

Description of Additional Supplementary Files

File name: Supplementary Data 1

Description: Source data file for graphs in Fig. 1
